# Determinants of patient survival during the 2014 Ebola Virus Disease outbreak in Bong County, Liberia

**DOI:** 10.1186/s41256-016-0005-8

**Published:** 2016-06-23

**Authors:** Thomas A. Weppelmann, Bangure Donewell, Ubydul Haque, Wenbiao Hu, Ricardo J. Soares Magalhaes, Mutaawe Lubogo, Lucas Godbless, Sasita Shabani, Justin Maeda, Herilinda Temba, Theophil C. Malibiche, Naod Berhanu, Wenyi Zhang, Luke Bawo

**Affiliations:** 1 Department of Environmental and Global Health, University of Florida, Gainesville, FL USA; 2 Emerging Pathogens Institute, University of Florida, 2055 Mowry Rd, Gainesville, FL USA; 3 African Union Support to Ebola Outbreak in West Africa (ASEOWA), Monrovia, Liberia; 4 Department of Geography, University of Florida, Gainesville, FL USA; 5 School of Public Health and Social Work, Queensland University of Technology, Brisbane, Australia; 6 School of Veterinary Science, The University of Queensland, Brisbane, Australia; 7 Children’s Health Research Centre, The University of Queensland, St Lucia, Australia; 8 Institute of Disease Control and Prevention, Academy of Military Medical Science, Beijing, People’s Republic of China; 9 Liberian Ministry of Health and Social Work (MOHSW), Monrovia, Liberia

## Abstract

**Background:**

The unprecedented size of the 2014 Ebola Virus Disease (EVD) outbreak in West Africa has allowed for a more extensive characterization of the clinical presentation and management of this disease. In this study, we report the trends in morbidity, mortality, and determinants of patient survival as EVD spread into Bong County, Liberia.

**Methods:**

An analysis of suspected, probable, or confirmed cases of EVD (*n* = 607) reported to the Liberian Ministry of Health and Social Welfare (MOHSW) between March 23^rd^ and December 31^st^ 2014 was conducted. The likelihood of infection given exposure factors was determined using logistic regression in individuals with a definitive diagnosis by RT-PCR (*n* = 321). The risk of short-term mortality (30 days) given demographic factors, clinical symptoms, and highest level of treatment received was assessed with Cox regression and survival analyses (*n* = 391).

**Results:**

The overall mortality rate was 53.5 % (95 % CI: 49 %, 58 %) and decreased as access to medical treatment increased. Those who reported contact with another EVD case were more likely to be infected (OR: 5.7), as were those who attended a funeral (OR: 3.9). Mortality increased with age (*P* < 0.001) and was higher in males compared to females (*P* =0.006). Fever (HR: 6.63), vomiting (HR: 1.93), diarrhea (HR: 1.99), and unexplained bleeding (HR: 2.17) were associated with increased mortality. After adjusting for age, hospitalized patients had a 74 % reduction in the risk of short term mortality (*P* < 0.001 AHR: 0.26; 95 % CI AHR: 0.18, 0.37), compared to those not given medical intervention.

**Conclusion:**

Even treatment with only basic supportive care such as intravenous rehydration therapy was able to significantly improve patient survival in suspected, probable, or confirmed EVD cases.

## Background

Ebola virus disease (EVD) is a highly contagious, acute hemorrhagic fever that is often fatal if not treated [1, 2]. EVD can be caused by a number of species within the genus *Ebolavirus*, in the family *Filoviridae*, which include: Taï Forest virus (TAFV), Reston virus (RESTV), Sudan virus (SUDV), Bundibugyo virus (BDBV), and Zaire Ebola virus (EBOV) [3]. The Ebola virus (EBOV) was discovered in 1976 during an outbreak of a highly fatal hemorrhagic fever in Sudan and Zaire and named after a small river located near the village of Yambuku where the patient sample that led to its isolation was collected [4]. Zoonotic transmission resulting from contact with infected primates or bats is thought to be the origin for most EVD outbreaks in humans, which until recently were mostly restricted to rural villages [5, 6]. Once introduced into the human population, rapid human-to-human transmission is facilitated by direct contact between mucosal membranes or broken skin and the bodily fluids (blood, sweat, saliva, urine, or feces) of an infected person [7, 8]. After an incubation period ranging from 2 to 21 days, the clinical presentation of EVD begins with influenza-like symptoms including fever, myalgia, and chills along with diarrhea and vomiting that can progress to hemorrhagic complications, shock, and multiple organ failure resulting in death [9, 10].

The first case of the current Ebola epidemic was reported on March 21, 2014 in Guinea, West Africa [11]. Three days later, the Liberian Ministry of Health and Social Welfare (MOHSW) received the first case reports of EVD along the Liberian border with Guinea and was informed that six suspected cases had traveled from Guinea to the districts of Foya and Zorzor in Lofa County, Liberia for treatment [12]. Almost one year after the outbreak, the Liberian MOHSW had reported 9645 suspected, probable, or confirmed cases of EVD with 4252 deaths [13]. Recently, multiple epidemiological investigations have been published, however the associations between demographic factors, clinical symptoms, and mortality from EVD were made using only the information available from the beginning of the outbreak [14–16]. The goal of this study was to characterize the trends in morbidity, mortality, and survival until the end of 2014 by using detailed epidemiological data from Bong, County Liberia. This report provides important perspectives on the demographic and clinical determinants of mortality and survival of suspected, probable, and confirmed EVD cases, as the Ebola outbreak spread rapidly across the border from Guinea into Liberia.

## Methods

### Study population, data collection, and ethics statement

**Fig. 1:**
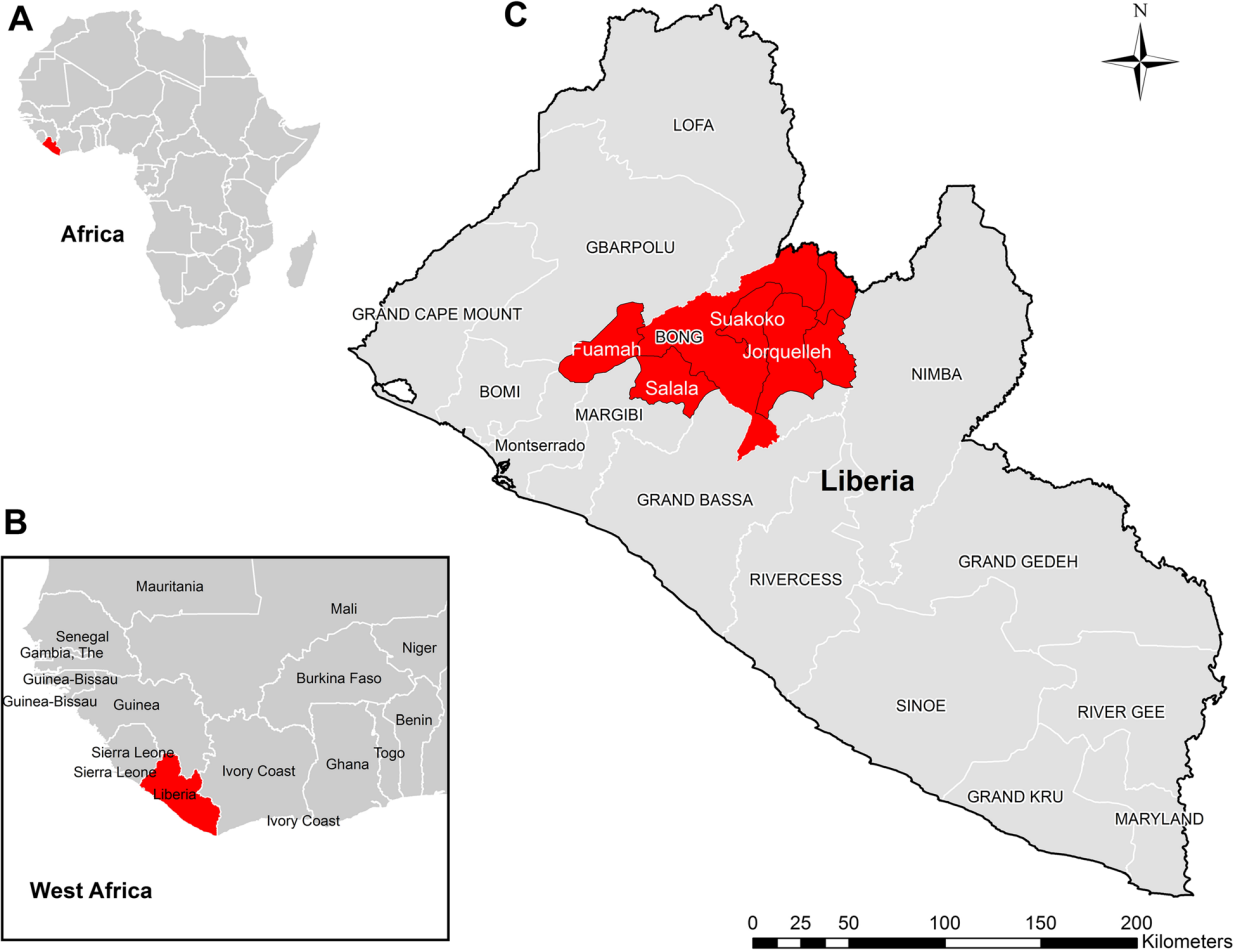
Map of Liberia with the location of suspected, probable, or confirmed EVD cases analyzed in this study. A map is presented detailing the location of Liberia within the continent of Africa (**a**), Liberia’s location within the West African coastline (**b**), and the locations of the study districts in Bong county (**c**) including the major enrollment sites of Fuamah (*n* = 101), Salala (*n* = 82), Suakoko (*n* = 98), and Jorquelleh (*n* = 200); along with the minor enrollment sites from the surrounding districts in red (*n* = 126)

Bong County is located in the north-central area of Liberia (Fig. 1). It is 1 of 15 counties in Liberia, covering 8772 km^2^ with a population of over 300,000 people. It has 37 health facilities including three hospitals: Phebe Hospital in Suakoko District, Bong Mines Hospital in Fuahmah District, and Dumbar Hospital in Jorquelleh District [17]. Bong County was selected because of its location adjacent to Lofa County, where the EVD outbreak in Liberia was suspected to originate and also because of the availability of highly detailed epidemiological data for EVD cases. Individual daily records of suspected, probable, and confirmed cases of EVD reported in Bong County from March 21^st^ to December 31^st^, 2014 were obtained from the Liberian Ministry of Health and Social Work (MOHSW). The date of onset of EVD symptoms, observed clinical manifestations, hospital admittance, contact history, and funeral attendance were collected along with demographic information such as age, sex, and occupation (healthcare worker or not) using a standardized case investigation form for all persons of interest suspected of EVD. All data was de-identified by the Liberian MOHSW by the removal of personal information or unique identifiers and given to the African Union Support to Ebola Outbreak in West Africa (ASEOWA) for analyses of the trends in morbidity, mortality, and survival in Bong County. The Liberian MOHSW ethics committee and the ASEOWA both approved the de-identification, release, and subsequent analysis of this data, which was collected during case management of EVD patients.

### EVD case definitions and treatment level classifications

Early diagnosis of EVD can be difficult given that the early symptoms are nonspecific and often seen in patients with more commonly occurring diseases, such as malaria and typhoid fever [8]. Using Word Health Organization (WHO) recommendations for EVD adapted for the West African outbreak [18], all reported persons of interest were classified as suspected (patients presenting with fever and/or three additional symptoms such as diarrhea, vomiting, or unexplained bleeding), probable (patients suspected of having EVD and reported contact history with another probable or confirmed EVD case), and confirmed (EVD diagnosis confirmed by laboratory methods). Specimens were collected from suspected and probable EVD cases for laboratory confirmation when resources were available. Whole blood was collected from 321 suspected or probable EVD cases for diagnostic testing using reverse transcriptase polymerase chain reaction (RT-PCR). EBOV nucleoprotein (EBOVNP) and glycoprotein (EBOVGP) genes were detected by real-time quantitative RT-PCR with oligonucleotide probes in a similar manner as previously described [19]. All reported suspected, probable, or confirmed cases were also classified by the highest level of treatment that they received, which included three levels 1) Isolation – patients isolated inside their residence or domicile with no formal treatment, 2) Quarantine – patients quarantined for observation and/or specimen collection with minimal treatment including oral rehydration salts, or 3) Hospitalization – patients hospitalized in community health center or medical facility with medical intervention consisting of intravenous fluids including Ringer’s lactate or glucose Ringer’s solution, metoclopramide or omeprazole to control vomiting, and/or antibiotics such as ciprofloxacin.

### Statistical analyses

**Fig. 2:**
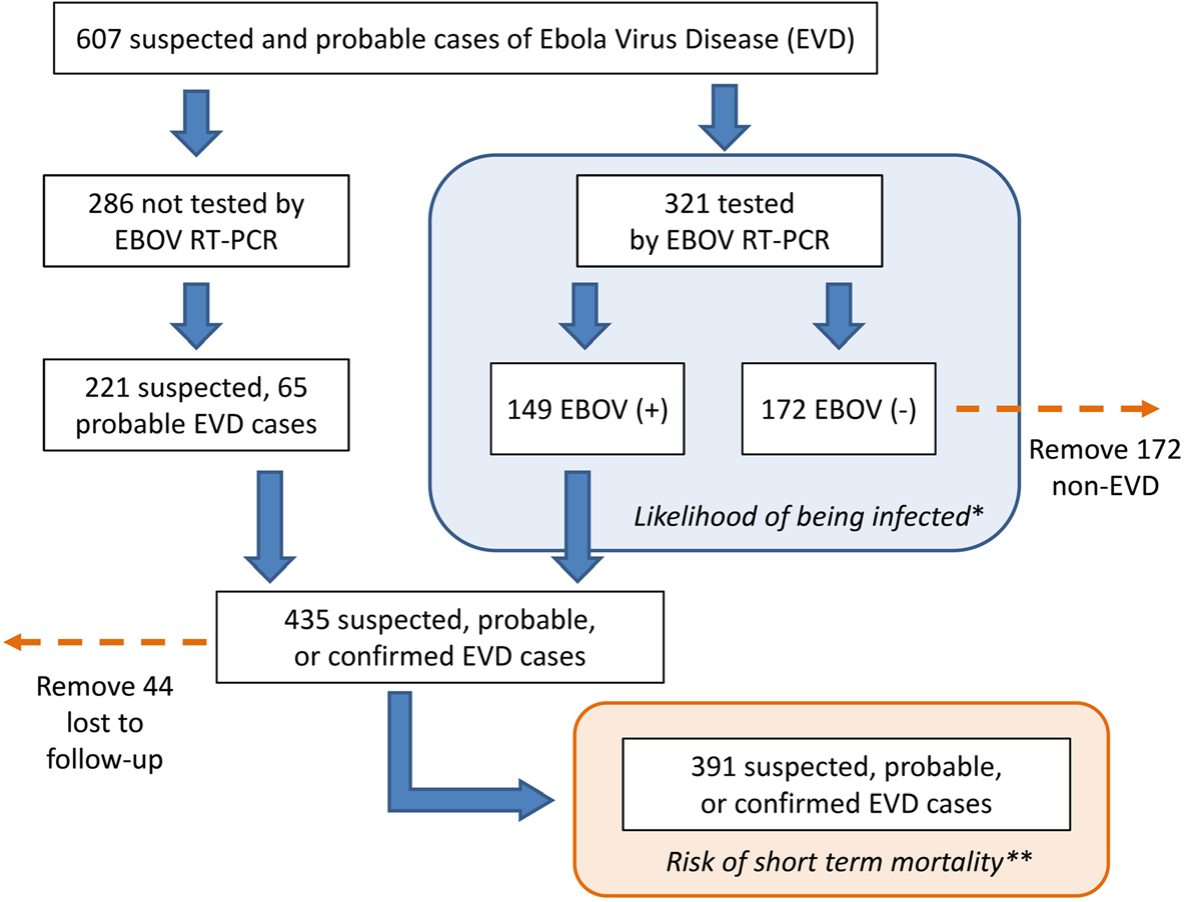
Number of suspected, probable, or confirmed cases of EVD. After the identification of persons of interest (*n* = 607), suspected and probable cases were subjected to diagnostic testing with RT-PCR for EBOV when available (*n* = 321). For those with test results, the likelihood of being RT-PCR positive given demographic and exposure related factors was assessed using simple logistic regression (blue shaded polygon*); the 172 RT-PCR negatives were excluded from further analysis. Cases confirmed by RT-PCR (*n* = 149) and suspected and probable EVD cases not tested by RT-PCR (*n* = 286) were submitted to review of survival histories for completeness, excluding 44 that were lost to follow-up at 30 days. This left 391 suspected, probable, or confirmed EVD cases for survival analysis using Cox proportional hazards regression and Kaplan-Meier survival functions to assess the risk of short-term mortality within 30 days of symptom onset (orange shaded polygon**)

Of suspected and probable EVD cases that received a diagnosis by RT-PCR (*n* = 321) the likelihood of being infected for those that had reported contact history with another probable or confirmed case, attended a funeral, or self-reported as a healthcare worker was assessed using simple logistic regression. After exclusion of 172 patients with negative PCR results and 44 suspected or probable EVD cases that were lost to follow-up; detailed survival histories from the reported onset of EVD symptoms until time of death (within 30 days) were available for 391 of the suspected, probable, or confirmed EVD cases (Fig. 2). The association between age, sex, clinical symptoms, and highest level of care (isolation, quarantine, or hospitalization) and the time from onset of EVD symptoms to death was determined using Cox proportional hazards models with and without adjustment for age. The risk of short term mortality was expressed by hazard ratios (HR) and presented along with age-adjusted hazard ratios (AHR) and their respective 95 % confidence intervals and level of statistical significance in Table 1. The survival functions for the risk of short-term mortality (30 days) for the same covariates at the Cox proportional hazards models were assessed using the Kaplan-Meier survival function. The age of suspected, probable, and confirmed EVD cases was not normally distributed and was divided into the following five approximately equal groups: >20 years (group 1, *n* = 74), 20 to 30 years (group 2, *n* = 72), 30 to 40 years (group 3, *n* = 67), 40 to 50 years (group 4, *n* = 79), and >50 years (group 5, *n* = 82). A log-rank test was used to assess the equality of survivor functions for the corresponding Kaplan-Meier survival functions.

**Table 1:** Risk of short-term mortality in suspected, probable, or confirmed EVD cases

Demographic factors^a^	HR^b^	*P* value	95 % Conf. Int.		AHR^c^	*P* value	95 % Conf. Int.	
age (years)	1.02	< 0.001	1.01	1.02	–	–	–	–
sex	1.51	0.007	1.12	2.03	1.52	0.006	1.13	2.05
Clinical symptoms	HR	*P* value	95 % Conf. Int.		*AHR*	*P* value	95 % Conf. Int.	
fever	6.63	0.001	2.12	20.76	2.50	0.116	0.80	7.83
vomit/nausea	1.93	< 0.001	1.35	2.77	1.58	0.013	1.10	2.26
diarrhea	1.99	< 0.001	1.42	2.77	1.66	0.003	1.19	2.32
fatigue	1.58	0.027	1.05	2.38	1.23	0.331	0.81	1.85
abdominal pain	1.26	0.120	0.94	1.70	1.18	0.273	0.88	1.59
muscle pain	1.08	0.613	0.79	1.48	0.85	0.307	0.62	1.16
joint pain	1.38	0.032	1.03	1.86	1.15	0.361	0.85	1.55
headache	0.95	0.755	0.71	1.28	0.82	0.188	0.61	1.10
difficulty swallowing	1.17	0.437	0.79	1.74	1.11	0.620	0.74	1.64
unexplained bleeding	2.17	0.013	1.18	3.99	2.327	0.007	1.26	4.30
number of symptoms	1.08	0.009	1.02	1.15	1.02	0.549	0.95	1.09
Treatment given^d^	HR	*P* value	95 % Conf. Int.		*AHR*	*P* value	95 % Conf. Int.	
isolation (*n* = 159)	ref	–	–	–	ref	–	–	–
quarantine (*n* = 70)	0.46	< 0.001	0.31	0.69	0.70	0.081	0.47	1.05
hospitalization (*n* = 162)	0.25	< 0.001	0.18	0.35	0.26	< 0.001	0.18	0.37

^a^216 males and 172 females were used (*n* = 388) with females as the reference group, for age seventeen records did not include the participant age in the case reporting form (*n* = 374)

^b^Hazard ratios (HR) determined by Cox regression

^c^Adjusted hazard ratio (AHR) determined by Cox regression adjusting for age in years

^d^Treatment given was stratified into three levels of care; with quarantine and hospitalization compared to those who were isolated (reference group)

The risk of short term mortality (within 30 days of EVD symptom onset) in suspected, probable, or confirmed EVD patients was determined by Cox proportional hazard models using demographic factors, clinical symptoms, and highest level of treatment received. The risk of mortality is presented as a hazard ratio (HR) with and without age adjustment (AHR), with *P* values and 95 % confidence intervals for the hazard ratios. Some demographic data was missing from the case forms, reducing the 391 survival histories to 374 for age and 388 for sex; consequently the AHR only include 374 people

## Results

### Trends in morbidity and mortality of EVD

**Fig. 3:**
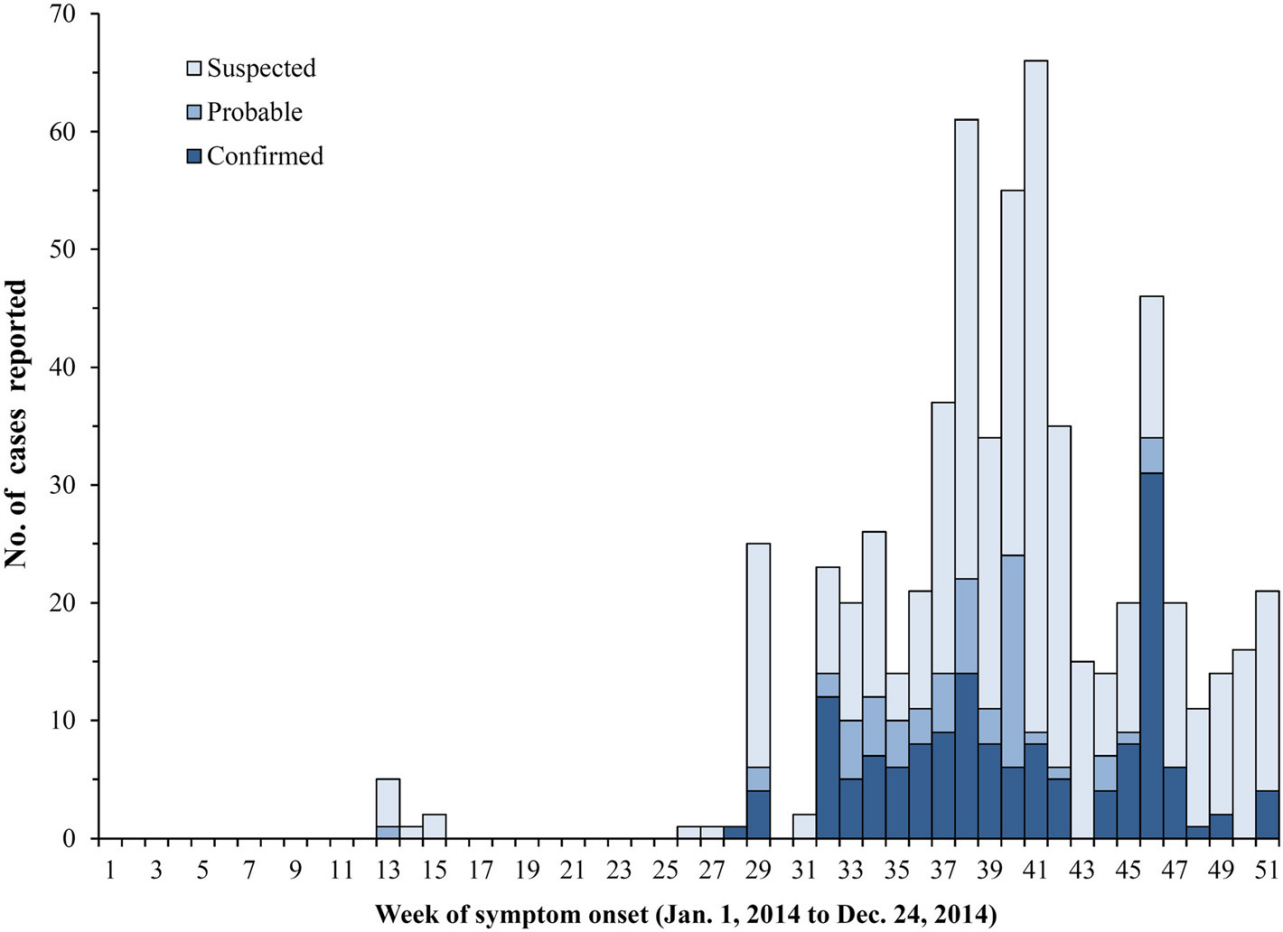
Number of suspected, probable, or confirmed cases of EVD. The numbers of suspected (gray), probable (light blue), or confirmed (navy) EVD cases reported from healthcare centers in Bong County, Liberia are presented by epidemiological week from Jan 1, 2014 to December 24, 2014 (weeks 1–51)

**Fig. 4:**
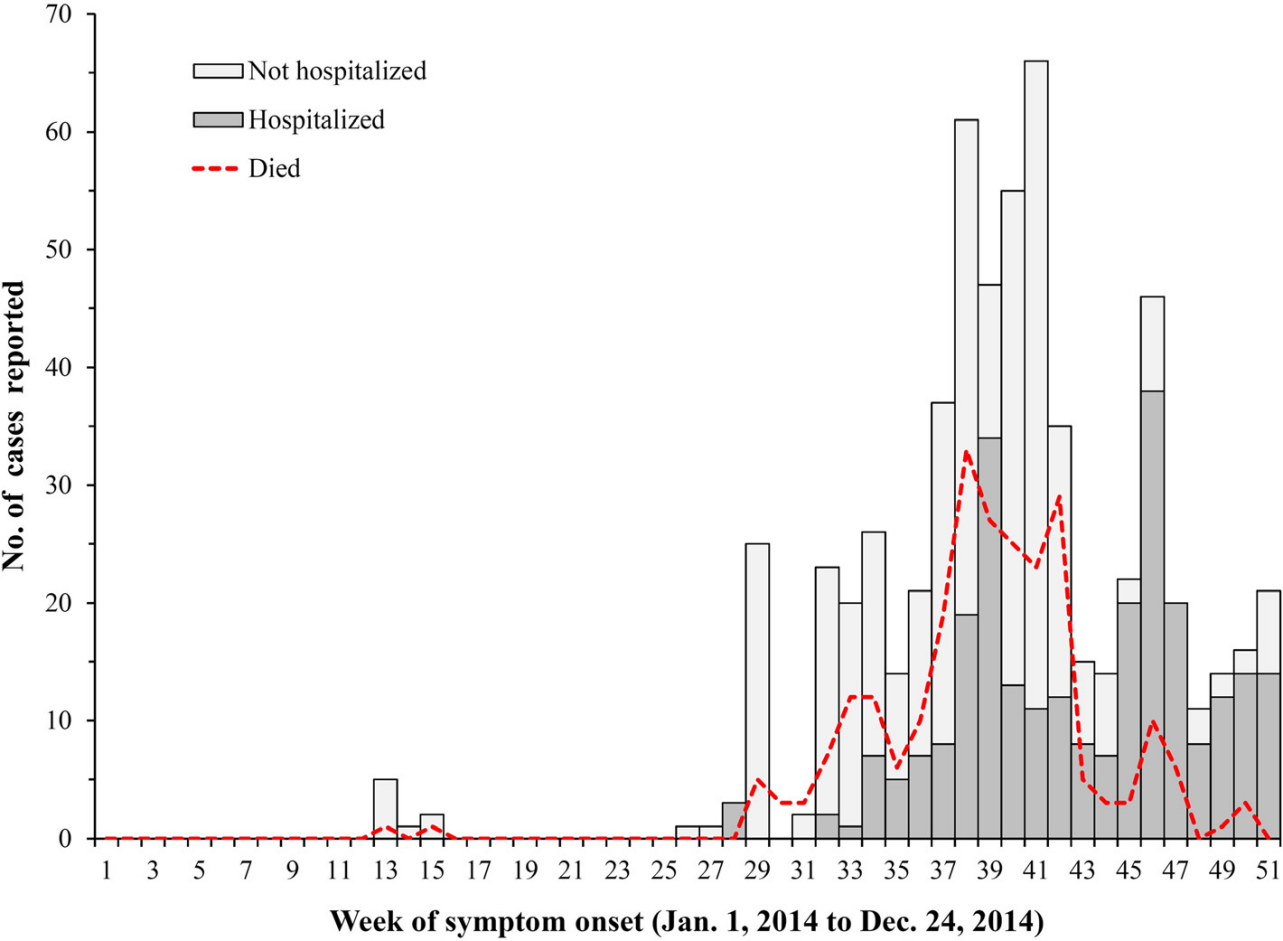
Number of reported suspected, probable, or confirmed EVD cases that were hospitalized or resulted in fatality. The numbers of suspected, probable, or confirmed EVD cases that were hospitalized (gray), quarantined or isolated (light gray), or those who died (red dotted line) from health care centers in Bong County, Liberia are presented by epidemiological week from Jan 1, 2014 to December 24, 2014 (weeks 1–51)

A total of 607 suspected or probable cases of EVD were reported to the Liberian MOHSW between March 21^st^ and December 31^st^. Suspected, probable, and confirmed EVD cases were aggregated by week and plotted as an epidemic curve, along with the proportion of those hospitalized and the mortality rate by week. After an initial report of EVD in March followed by 10 weeks with no reported cases, EVD started to spread in epidemic form in Bong County by August; with incidence peaking between September and November 2014 (weeks 37–46; Fig. 3). Approximately 55.2 % of the suspected, probable, or confirmed cases (*n* = 428) were males, with an average age of 36.3 years (Std. deviation: 19.4 years; IQR = 24–48 years). The percentage of reported EVD symptoms were as follows: fever (91.4 %), vomiting and nausea (70.1 %), diarrhea (65.0 %), fatigue (80.4 %), abdominal pain (59.1 %), muscle pain (68.5 %), joint pain (57.7 %), headache (43.7 %), difficulty swallowing (13.8 %), unexplained bleeding (4.7 %), with patients reporting on average six of the aforementioned symptoms (IQR = 5–7). From the 21^st^ of March to the 31^st^ of December 2014, 229 of the 428 suspected, probable, or confirmed EVD cases died, giving a mortality rate (MR) of 53.5 % (95 % CI MR: 49 %, 58 %). A notable feature of the outbreak in Bong County was the increase in the proportion of cases that were hospitalized as more resources were allocated to mitigate the spread of EVD throughout Liberia and West Africa, which resulted in a decrease in the number of deaths by late November and December (Fig. 4). Among suspected, probable, or confirmed EVD cases that were hospitalized, the MR was 30.0 % (95 % CI MR: 23 %, 37 %), compared to a MR of 73.7 % (95 % CI MR: 67.7, 79.8) for those not admitted to a hospital or treatment center and given medical interventions.

### Risk factors for infection among suspected or probable cases tested by PCR

Multiple exposure-related factors were associated with increased likelihood of EVD infection in person of interest. Of all persons of interest screened by PCR, 46.4 % (149/321) were positive for EBOVNP/EBOVGP; compared to 74.8 % (74/99) of patients who reported contact history with another suspected, probable, or confirmed EVD case and 75 % (12/16) who reported recently attending a funeral of a suspected, probable, or confirmed EVD case. Among the suspected or probable cases tested, there was over a 5 fold increase in the likelihood of being infected for those with previous contact history with another probable or confirmed case of EVD (OR: 5.7; 95 % CI: 3.3, 10) and an almost 4 fold increase in those who reported recently attending a funeral of a probable or confirmed EVD case (OR: 3.9; 95 % CI: 1.2, 12.3). The sample only contained 13 participants that identified themselves as healthcare workers, however all healthcare workers (11/11) screened by PCR were positive for EBOVNP/EBOVGP. Among suspected and probable cases tested, the likelihood of having a positive PCR result was slightly higher, but not statistically significant (*P* = 0.082) with increasing age (OR: 1.012; 95 % CI: 0.998, 1.026) and not significantly different by gender (*P* = 0.147).

### Risk of mortality in suspected, probable, or confirmed EVD cases

**Fig. 5:**
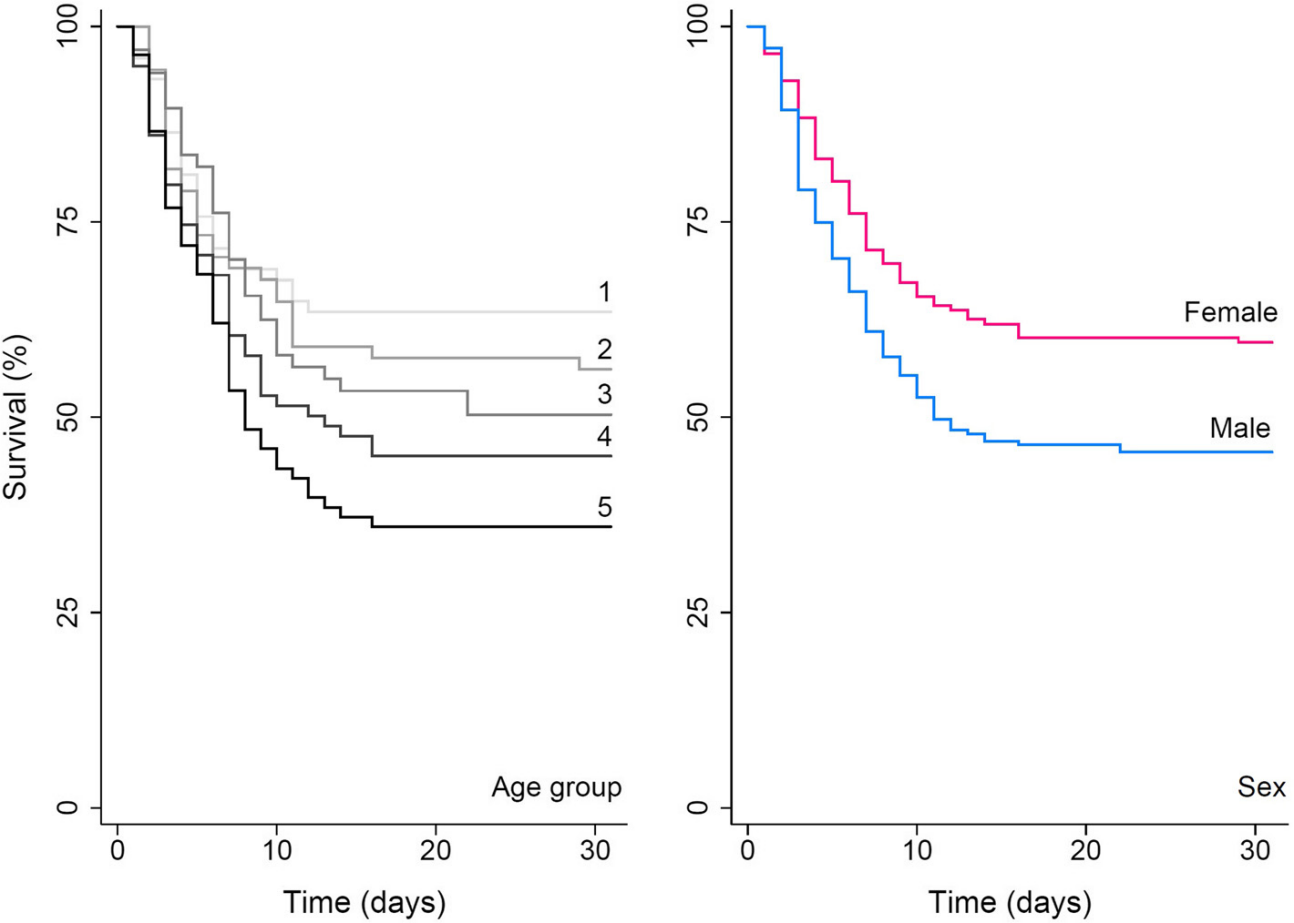
Survival of suspected, probable, or confirmed EVD cases by age and sex. The Kaplan-Meier survival functions for the time period 30 days after the onset of EVD symptoms is presented for suspected, probable, or confirmed EVD cases. Survival by age group (left panel) was stratification into the following age groups: >20 years (group 1, *n* = 74), 20 to 30 years (group 2, *n* = 72), 30 to 40 years (group 3, *n* = 67), 40 to 50 years (group 4, *n* = 79), and >50 years (group 5, *n* = 82), which appear as a gradient of gray lines from lightest to darkest (groups 1 to 5, respectively). Survival by sex (right panel) included 216 males (*blue line*) and 172 females (*pink line*). The equality of survival functions assessed by the log-rank test was significantly different by age group (*P* = 0.01) and sex (*P* = 0.002)

**Fig. 6:**
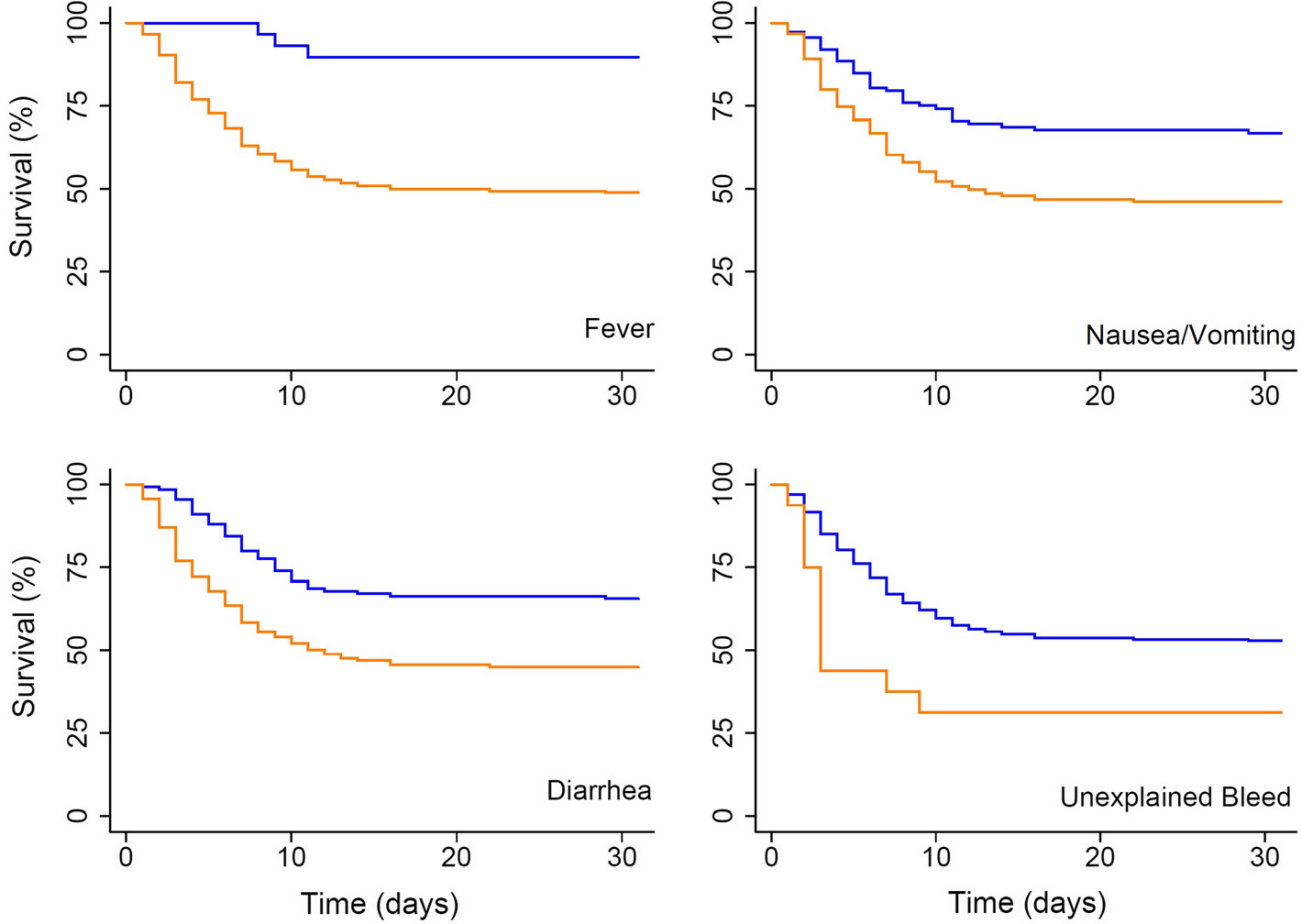
Survival of suspected, probable, or confirmed cases of EVD by clinical symptoms. The Kaplan-Meier survival functions for the time period 30 days after the onset of EVD symptoms is presented for suspected, probable, or confirmed EVD cases. Survival by clinical symptoms of fever, nausea/vomiting, diarrhea, and unexplained bleeding are presented as those with the symptom (*orange*) compared to those without the symptom (*blue*). The equality of survival functions assessed by log-rank tests was significantly different for those with fever (*P* < 0.001), nausea/vomiting (*P* > 0.001), diarrhea (*P* < 0.001), and unexplained bleeding (*P* = 0.009)

**Fig. 7:**
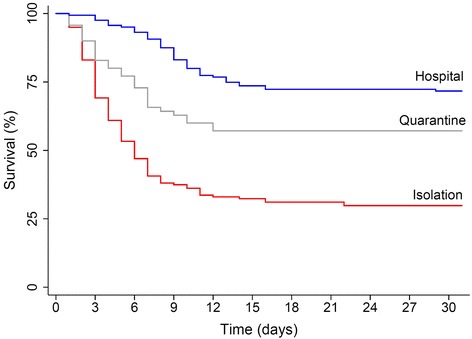
Survival of suspected, probable, or confirmed cases of EVD by treatment given. The Kaplan-Meier survival functions for the time period including 30 days after the reported onset of symptoms is presented for suspected, probable, or confirmed EVD cases. Survival functions are stratified by the highest level of treatment received, which was categorized by those who were hospitalized (*blue line*, *n* = 162), those who were quarantined with other individuals suspected of having EVD (*gray line*, *n* = 70), and those who were isolated (*red line*, *n* = 159). The equality of survival functions assessed by log-rank tests was significantly different by the highest level of treatment given (*P* < 0.001)

The risk of short term mortality in suspected, probable, or confirmed cases of EVD given demographic factors, clinical symptoms, and treatment is presented in Table 1. The hazard ratio was significantly different by age (*P* < 0.001), where each additional 10 year increase in age was associated with a 17.8 % increase in the likelihood of mortality (HR: 1.18; 95 % CI: 1.09, 1.27). Gender was also significantly associated (*P* = 0.007) with increased mortality, where the risk of short term mortality for males was 1.51 times as large compared to females (95 % CI HR: 1.12, 2.03), even after adjustment for age. The Kaplan-Meier survival functions for different age groups and sex are presented in Fig. 5, which gave significantly different survival functions for age group (*P* = 0.01) and sex (*P* = 0.002). Multiple clinical symptoms were associated with increased mortality in suspected, probable, or confirmed EVD cases including: fever (HR: 6.63; 95 % CI: 2.12, 20.8), vomiting (HR: 1.93; 95 % CI: 1.35, 2.77), diarrhea (HR: 1.99; 95 % CI: 1.42, 2.77), and unexplained bleeding (HR: 2.17; 95 % CI: 1.18, 3.99). The Kaplan-Meier survival functions for different clinical symptoms are presented in Fig. 6 which gave significantly different survival functions for the those presenting with fever (*P* < 0.001), vomiting (*P* < 0.001), diarrhea (*P* < 0.001), and unexplained bleeding (*P* = 0.009). The survival of suspected, probable, or confirmed EVD cases from the reported onset of symptoms for patients that were hospitalized (*n* = 162), individuals who were quarantined awaiting admission to treatment centers (*n* = 70), and those who were isolated in their domicile (*n* = 159) revealed significant differences in the survival functions (*P* < 0.001; Fig. 7). Compared to isolated individuals, those who were quarantined had a 54 % lower risk of short term mortality (HR: 0.46; 95 % CI HR: 0.31, 0.69), and those who were hospitalized had a 75 % lower risk of short term mortality (HR: 0.25; 95 % CI: 0.18, 0.35). After adjustment for the effect of age, a comparison to individuals who were isolated revealed that those who were quarantined had a 30 % lower risk of short term mortality (AHR: 0.70; 95 % CI AHR: 0.47, 1.0) and those who were hospitalized remained at approximately 75 % lower risk of short term mortality (AHR: 0.26; 95 % CI AHR: 0.18, 0.37).

## Discussion

Almost a year after the Ebola outbreak began, 25,030 suspected or probable EVD cases have been reported in Guinea, Sierra Leone, and Liberia, with 14,753 confirmed by laboratory methods and 10,398 resulting in death; making this the largest Ebola outbreak in history [20]. While the number of reported cases continues to decline, there is still an ongoing epidemic of EVD in parts of West Africa [21]. This report provides one of the first detailed epidemiological descriptions of the initial spread of the Ebola epidemic across the border from Guinea into Liberia. The epidemic grew rapidly from the introduction to the neighboring Lofa County in March, peaking in Bong County from September through October, before declining again in late December 2014 (Fig. 3). Along with lower numbers of reported cases in late December, the mortality rate in suspected, probable, or confirmed EVD cases drastically decreased as more patients were hospitalized (Fig. 4). Transmission between healthcare workers and those receiving care, as well as those who attended funerals of confirmed or probable EVD cases were implicated as important factors that contributed to the early spread of the epidemic, with high rates of transmission within families and communities [22, 23]. Though only a limited number of suspected, probable, or confirmed EVD cases reported attending a funeral or being a healthcare worker, the rates of infection in these groups was much higher than in the other persons of interested reported to Bong County Ebola treatment centers. As reported elsewhere in West Africa, the mortality rate in healthcare workers was also higher compared to the other population members in this study (MR 76.9 % vs 53.5 %) [24].

The rates of mortality in suspected, probable, or confirmed EVD patients from Bong County were very similar to other populations in Liberia and West Africa, which had an average rate of 41.5 % and most estimates ranging between 30 and 60 % [6, 25, 26]. The association of older age with increased mortality found in this sample was also identified in EVD patients in West Africa [16, 27, 28], although the differences in mortality among males and females from this study were likely confounded by hospitalization rates. Males were significantly less likely (*P* = 0.009) to be hospitalized compared to females (OR: 0.58; 95 % CI OR: 0.38, 0.87), and after adjustment for age and hospitalization, the association between male gender and increased mortality was no longer significant (*P* = 0.106). Increased age was also associated with decreased likelihood of hospitalization (OR: 0.98, 95 % CI OR: 0.97, 0.99); though the association between age and reduced mortality was still significant and almost identical when adjusting for hospitalization. As in other studies, clinical symptoms included fever, diarrhea, nausea and vomiting, fatigue, joint pain, and unexplained bleeding, all of which were found to be associated with increased risk of mortality in suspected, probable, or confirmed cases of EVD [16, 26, 29]. Given the associations identified in this study between increased hospitalization and decreased mortality, the level of treatment given could explain the differences in the mortality rates reported in other West Africa countries. In many of the healthcare facilities treating patients in Bong County only basic supportive care was available; mostly in the form of oral and intravenous rehydration solutions, antiemetic agents, and antibiotics. Since less than 5 % of the suspected, probable, and confirmed EVD cases in this sample experienced unexplained bleeding and hemorrhage, the increase in survival in hospitalized patients was likely due to prevention of intravascular volume depletion and replacement of electrolytes, which greatly reduces the potential for complications arising from hypovolemic shock [30, 31].

### Limitations

Though this study provides some valuable insights into the trends in morbidity, mortality, and determinants of patient survival it is not without limitations. The likelihood of infection given exposure factors was not designed to assess the risks faced by the general population, but only to estimate the likelihood of being infected with EVD for persons of interest in Bong County. In this study, only individuals with negative RT-PCR results were excluded, which likely included some suspected and probable EVD cases that did not have EVD. However, the ability to diagnose EVD by PCR during the Liberian outbreak was limited, and we believe the description of the survival of suspected and probable EVD patients in this study is potentially useful to the majority of treatment centers that lack the ability to definitively diagnose EVD. As such, the potential selection bias on the mortality rates and survival in suspected, probable, or confirmed EVD cases was unable to be determined. Nonetheless, the mortality rates from this study were in agreement with those observed elsewhere in Liberia and provide valuable information on the survival of suspected, probable, and confirmed EVD cases over the course of the 2014 outbreak. Likewise, it is possible that some suspected, probable, or confirmed EVD cases were isolated or quarantined prior to treatment, or that the level of treatment at hospitals differed; making the estimates of the effect of medical intervention unequal among all suspected, probable, or confirmed EVD cases.

## Conclusion

Though the unprecedented size of the current Ebola outbreak in West Africa has challenged the international community’s ability to respond to such an emergency, it has also provided unique opportunities to understand ways to control Ebola transmission and improve the clinical management of EVD. Despite the relatively high rates of mortality observed in this study, the marked increased in patient survival in suspected, probable, or confirmed EVD cases who were hospitalized is an encouraging finding. With the appropriate use of protective equipment to prevent the spread of EVD during treatment, these findings suggest that during an EVD epidemic, a significant reduction in mortality can be achieved with proper administration of even the most basic of supportive therapies.
